# Real-Time Detection of a Virus Using Detection Dogs

**DOI:** 10.3389/fvets.2015.00079

**Published:** 2016-01-08

**Authors:** T. Craig Angle, Thomas Passler, Paul L. Waggoner, Terrence D. Fischer, Bart Rogers, Patricia K. Galik, Herris S. Maxwell

**Affiliations:** ^1^Canine Performance Sciences Program, Auburn University, Auburn, AL, USA; ^2^Department of Clinical Sciences, Auburn University, Auburn, AL, USA; ^3^Animal Health Research, Auburn University, Auburn, AL, USA

**Keywords:** bovine viral diarrhea virus, dog, odor, virus detection, volatile organic compounds

## Abstract

Viral infections are ubiquitous in humans, animals, and plants. Real-time methods to identify viral infections are limited and do not exist for use in harsh or resource-constrained environments. Previous research identified that tissues produce unique volatile organic compounds (VOC) and demonstrated that VOC concentrations change during pathologic states, including infection, neoplasia, or metabolic disease. Patterns of VOC expression may be pathogen specific and may be associated with an odor that could be used for disease detection. We investigated the ability of two trained dogs to detect cell cultures infected with bovine viral diarrhea virus (BVDV) and to discriminate BVDV-infected cell cultures from uninfected cell cultures and from cell cultures infected with bovine herpes virus 1 (BHV 1) and bovine parainfluenza virus 3 (BPIV 3). Dogs were trained to recognize cell cultures infected with two different biotypes of BVDV propagated in Madin–Darby bovine kidney cells using one of three culture media. For detection trials, one target and seven distractors were presented on a scent wheel by a dog handler unaware of the location of targets and distractors. Detection of BVDV-infected cell cultures by Dog 1 had a diagnostic sensitivity of 0.850 (95% CI: 0.701–0.942), which was lower than Dog 2 (0.967, 95% CI: 0.837–0.994). Both dogs exhibited very high diagnostic specificity (0.981, 95% CI: 0.960–0.993) and (0.993, 95% CI: 0.975–0.999), respectively. These findings demonstrate that trained dogs can differentiate between cultured cells infected with BVDV, BHV1, and BPIV3 and are a realistic real-time mobile pathogen sensing technology for viral pathogens. The ability to discriminate between target and distractor samples plausibly results from expression of unique VOC patterns in virus-infected and -uninfected cells.

## Introduction

Globally, infectious diseases continue to be leading causes of morbidity and mortality. Efforts to control infectious disease in human, animal, and plant populations would benefit from real-time screening technologies, which could be effectively deployed in areas of strategic interest for pathogen transmission. Current surveillance methods rely on the collection of diagnostic samples from individuals or contaminated environments, transportation of samples to a laboratory, and subsequent laboratory testing to demonstrate the presence of the pathogen of interest, resulting in a significant delay in response times and containment efforts. Development of a sensitive, easily deployable real-time mobile pathogen sensing technology (RMST) would be useful in border security, public health, wildlife management, and agriculture to aid in the detection and containment of disease outbreaks and to prevent acts of bioterrorism and agro terrorism.

The abilities of trained dogs to identify odors associated with explosives and munitions in operational environments are superior to other currently available detection technologies. The highly sensitive canine olfactory sensory system can detect some target substances at concentrations as low as parts per trillion (1), three orders of magnitude more sensitive than available instruments which reliably identify substances at concentrations of parts per million or billion. Canine detection systems are inherently mobile and can trace an odor to its source. To be comparable to the detection ability of dogs, a detection system would need to be (1) extraordinarily sensitive, (2) mobile, and (3) able to move toward a target source. No currently available system meets those criteria. If dogs can be trained to detect odors associated with specific pathogens, canine-based RMST systems could provide a method for pathogen detection in frontline operational environments and complement the development of detection instrumentation for pathogen detection, analytical chemistry, and metabolomics.

### Pathogen Biomarkers for Scent Detection

Strong scientific evidence supports the release of stable volatile organic compounds (VOC) from tissues that are present in exhaled breath, urine, feces, and sweat. The VOC that are released are known as the volatilome (2). VOC are volatile at ambient temperatures, may be associated with an odor, and may provide the continuous signal needed for real-time detection (3).

Tissues infected with pathogens release unique volatile metabolic biomarkers, which become part of detectable VOC disease signatures (4, 5). Disease-specific VOC show potential for use in medical diagnosis and therapeutic monitoring (2). Multiple studies demonstrated that VOC patterns may be unique to a specific pathogen or an infection with a specific pathogen. In a review of 31 publications, Bos et al. (6) concluded that many pathogenic bacteria have distinct metabolisms that produce species-specific VOC and suggested that the presence of these VOC in patients indicated infection. In a cell culture model, Schivo et al. (7) demonstrated different VOC expression patterns in primary human tracheobronchial cells infected or uninfected with human rhinovirus. Aksenov et al. (8) determined that VOC produced by B lymphoblastoid cells following infection with three live influenza virus subtypes were unique for each virus subtype. In addition, Abd El Qader et al. (9) examined the VOC released from cultures of five viruses (influenza A, influenza B, adenovirus, respiratory syncitial virus, and parainfluenza 1 virus) and four bacteria (*Moraxella catarrhalis*, *Haemophilus influenza*, *Legionella pneumophila*, and *Mycoplasma pneumoniae*). The researchers detected 12 and 6 VOC that were associated with bacterial and viral growth and identified 2 VOC that were differentiated between bacterial and viral infection (9). Lastly, Mashir et al. (10) administered live attenuated H1N1 vaccine (FluMist^®^) to humans and demonstrated that exhaled breath VOC increased for 7 days after the vaccination. These studies suggest that unique VOC profiles associated with viral pathogens exist and that they may be detected in patients. If pathologic processes such as infections, neoplasia, and metabolic disorders influence the type, ratio, and strength of VOC emitted from a biological system, then unique VOC patterns may create a specific signature odor (11). Currently, VOC analysis requires expensive and sophisticated stationary analytical chemical instrumentation, such as gas chromatography–mass spectrometry.

### Dogs as a Pathogen and Disease Sensor

Trained dogs have consistently demonstrated value as sensitive real-time chemical sensing detectors for narcotic, explosive, and select biological targets. Similarly, dogs have been used to detect disease biomarkers in humans, including cancer and bacterial infections. Potential VOC biomarker concentrations are reported to be in the part per billion to part per trillion range for breath and part per million to part per billion range in blood and urine (12). Canids are capable of detecting some substances in concentrations as low as parts per trillion (1). Therefore, VOC biomarkers are within the detection range of the canine olfaction system. In some cases, dogs have been able to detect disease states in exhaled breath that contains the lowest known VOC concentrations. Sonoda et al. (13) trained a dog to detect human patients with colon cancer using samples of exhaled breath and watery stool. The dog’s sensitivity and specificity for cancer detection in breath samples were 0.91 and 0.99, respectively. The sensitivity and specificity for detection in stool samples were 0.97 and 0.99, respectively (13).

Another study demonstrated that the overall sensitivity of canine scent detection of lung cancer utilizing exhaled breath samples was 0.99, with a specificity of 0.99 (14). In the same report utilizing trained dogs to evaluate breath samples from breast cancer patients and controls, the sensitivity of detection was 0.88 and specificity was 0.98 (14). These reports suggest that VOC or similar compounds from diseased internal tissues are released externally and may be detected on the body or in the surrounding air with the aid of trained dogs with a high degree of diagnostic accuracy.

Pathogen detection methods utilizing the keen canine sense of smell may offer a viable option for developing a rapidly deployable disease screening tool and provide valuable information about a subject’s pathophysiological condition (3, 15). The use of trained detection dogs offers certain inherent advantages. Unlike a deployable instrument, dogs can examine thousands of samples or scan large surface areas efficiently, which is important in detecting pathogens in large herds of animals, crowds of people, objects (e.g., ships, airplanes, buildings), or areas of land. Diagnostic testing using laboratory instrumentation in an operational environment is often impeded by the lack of cleanliness, interference by air particulates, presence of non-target VOC produced by various substances in the environment, and constantly changing variables, such as temperature, humidity, wind, and thermal plumes. By contrast, purpose-bred detection dogs have a demonstrated ability to search for unique odor patterns and identify specific targets in field conditions amidst substantial “odor noise” (i.e., varied and/or strong odors). Although at least 381 unique VOC are emitted from human feces (2), a trained dog was able to detect *Clostridium difficile* in human stool samples (16). The dog detected *C*. *difficile* with high diagnostic sensitivity and specificity in stool samples and hospitalized patients, correctly identifying 25 of the 30 *C. difficile* cases and 265 of 270 control cases (16). This emphasizes the dog’s ability to detect a specific odor pattern among the myriad of odors from other bacteria, fungi, and viruses naturally present in feces.

There are no reported attempts to train dogs to detect viral pathogens. Detection of infection or disease by trained dogs could provide advantages over other VOC detection technologies such as mass spectrometry by providing a real-time binary response, avoiding the need for trained personnel in the processing and interpretation of mass spectrometry samples, and avoidance of testing-associated delays in response efforts. Dogs are mobile, adapted to difficult work environments, can track a plume of airborne target material to its source and can eliminate the need to collect and transport surface or air samples to a centralized laboratory. If dogs can be trained to locate target pathogens, they could be employed to detect pathogens or be deployed at strategic locations to prevent entry and transmission of disease.

The use of dogs to detect odors associated with viral infection and sensitivity and specificity of a canine detection model has not been described. The purpose of this study was to evaluate the ability of trained dogs to detect viral pathogen-associated odors in real-time, alert a handler to the presence of these pathogens, and discriminate those odors from those associated with other viral pathogens. Specifically, we examined the dog’s ability to identify bovine viral diarrhea virus (BVDV) infected cell cultures and to discriminate those cell cultures from those infected with other bovine viral pathogens.

## Materials and Methods

### Animals

Two healthy adult male Labrador Retrievers were trained to detect BVDV-infected cell cultures. The dogs were purpose-bred for detection work from a colony of detection dogs developed at the Auburn University Canine Performance Sciences Breeding Program. Each dog had over 3 years of operational experience as an explosives detection dog in our Canine Performance Sciences Research Program. The dogs were selected based on their previous experience as explosives detection dogs and because they had calculated and methodical microsearch techniques that are important for the detection of viral targets. The dogs received 2 months of proprietary viral target detection training prior to data collection. The dogs were trained by a Master Detection Dog Trainer who had over 35 years of experience training detection dogs. The dogs were trained in a Bio Safety Lab Level 2 (BSL2) during the training period and received up to 15–30 trials per day, 4–5 days/week. All activities for this project were approved by an Institutional Animal Care and Use Committee and a Biological Use Authorization was granted by an Institutional Biosafety Committee.

### Testing Apparatus

A 12 × 12 foot, climate and humidity controlled, indoor, BSL2 isolation room was used for scent testing. In the center of the room was a scent wheel with eight arms that are designed to each hold a small metal basket. For each trial, one target odor and seven distracting odors (or eight distracting odors for blank trials) (Table 1) were each placed in separate glass Petri dishes, covered by a mesh screen, and then individually placed in a metal basket, one per arm of the scent wheel. All target odors were randomly assigned a position (1–8) on the scent wheel. Dogs were brought into the room and allowed to search, starting at position 1 and working to position 8. When the dog found the target odor, it alerted by sitting, and was rewarded with a toy.

**Table 1:** **Target viruses (BVDV AU526 and BVDV NADL) and distractor viruses (BHV 1 and BPI 3) were propagated on Madin–Darby bovine kidney (MDBK) cells using one of three media**.

Targets	Distractors
1A (*n* = 19): BVDV AU526 **(Non-cytopathic)** + MDBK + **EQS** (SPT = 3)	1B = MDBK + **EQS**
2A (*n* = 21): BVDV AU526 **(Non-cytopathic)** + MDBK + **FBS** (SPT = 1)	2B = MDBK + **FBS**
3A (*n* = 15): BVDV AU526 **(Non-cytopathic)** + MDBK + EQS + **Gentamicin** (SPT = 2)	3B = MDBK + **EQS** + **Gentamicin**
4A (*n* = 2): BVDV NADL **(Cytopathic)** + MDBK + **EQS** (SPT = 0)	7A = **BHV-1 (Cytopathic)** + MDBK + **EQS**
5A (*n* = 2): BVDV NADL **(Cytopathic)** + MDBK + FBS (SPT = 1)	8A = **BHV-1(Cytopathic)** + MDBK + **FBS**
6A (*n* = 5): BVDV NADL **(Cytopathic)** + MDBK + EQS + **Gentamicin** (SPT = 0)	9A = **BHV-1 (Cytopathic)** + MDBK + **Gentamicin**
	10A = **BPIV-3 (Cytopathic)** + MDBK + **EQS**
	11A = **BPIV-3 (Cytopathic)** + MDBK + **FBS**
	12A = **BPIV-3 (Cytopathic)** + MDBK + **EQS** + **Gentamicin**

*While cell culture media 1, 3, 4, and 6 contained equine serum (EQS), fetal bovine serum (FBS) was used in the preparation of medium 2 and 5. Cell culture medium 3 and 6 were prepared using the antibiotic gentamicin as an additional distractor. The n values represent the total number of samples used in the target column and the SPT values equal the total number of search past targets for each sample. The underlined text emphasizes the different characteristics of the two strains of BVDV that were used; the text in bold emphasizes differences among the three types of media*.

### Targets and Distractors

Bovine viral diarrhea virus (BVDV) was chosen as the target virus, because it provides a pathogen model that has been extensively studied and is easily propagated in different types of cells and media and is not pathogenic to humans or dogs. A non-cytopathic strain of BVDV (1b AU526) and a cytopathic strain of BVDV (1a NADL) were used as target viruses. The BVDV targets were propagated in Madin–Darby bovine kidney (MDBK) cells using one of three variations of culture media. The dogs were presented with 0.5 ml of target sample containing either cytopathic or non-cytopathic BVDV [1 × 10^5^–1 × 10^6^ CCID_50_ (cell culture infective doses, 50% endpoint)/ml]. The distractor viruses used in this study were bovine herpesvirus 1 (BHV 1) Colorado strain and bovine parainfluenza virus 3 (BPIV 3) SF/4. Preparation of all samples was performed by the same laboratory technician wearing identical nitrile gloves to prevent odor differences among samples caused by differences in sample handling. Target and distractor viruses were propagated in 75 cm^2^ cell culture flask that had been seeded with MDBK cells 24 h earlier. Three hundred microliters of stock virus were added to each flask in 3 ml of media. Cell culture media contained purified water, Minimal Essential Media with Earle’s salts (GIBCO^®^ MEM, 10×, 11430, Thermo Fisher Scientific, Life Technologies, Grand Island, NY, USA), l-glutamine [GIBCO^®^ l-glutamine (200 mM), Thermo Fisher Scientific, Life Technologies, Grand Island, NY, USA], PSF [GIBCO^®^ Antibiotic-Antimycotic (100×), Thermo Fisher Scientific, Life Technologies, Grand Island, NY, USA], sodium bicarbonate (GIBCO^®^ Sodium Bicarbonate 7.5% solution, Thermo Fisher Scientific, Life Technologies, Grand Island, NY, USA), and serum. Three different cell culture media were prepared that differed either in the type of serum added (equine serum (1 and 4) or fetal bovine serum (FBS) (2 and 5)), or contained gentamicin as an additional antibiotic (3 and 6). Following 1 h of adsorption, 20 ml of additional medium was added. Flasks were incubated until cytopathic effect in approximately 60% of cells was observed (BVDV 1 NADL, BHV 1, and BPI 3) or for 3 days when propagating the non-cytopathic BVDV AU526. Virus was harvested by a single freeze–thaw cycle by placing the flask in a −80°C freezer. Following thawing, contents of each flask were aliquoted into plastic snap-top tubes and stored (−80°C) until needed. Virus-free distractor samples underwent identical preparation including 24 h incubation prior to addition of 20 ml of media, incubation for 2–3 days following addition of media, and submission to a single freeze–thaw cycle prior to aliquoting and storage. For training and testing of dogs, 0.5 ml of each sample was placed into glass Petri dishes.

Distractors, or “non-target odors,” are used to provide scents that are similar to or slightly different than the positive target to ensure that the dog is truly indicating on the positive target. Distractors in this study are listed in Table 1.

The duration of exposure to target and non-target odors was very short, typically <0.25 s (i.e., the amount of time needed to sniff a basket), and determined by individual dog search behavior; dogs were never manually forced to sample the vapor from any target source (i.e., the dog is always free to repel from the source of the odor). The dogs typically searched all eight scent wheel positions in 3.5–4.0 s. Targets and distractors were presented in a manner to prevent dogs from physically contacting or ingesting the sample.

Extensive efforts were made to reduce confounding factors that could lead to false positive results or inflated measures of detection performance unrelated to detection/non-detection of the virus odor outside that of the dog smelling the target virus odor. Only one target sample was used per trial and its position among the eight arms of the scent wheel on each trail was randomly assigned. One hundred percent of targets and distractors and their holding containers were changed after each trial. Baskets, basket holders, scent wheel apparatus, and Petri dishes were only handled using nitrile gloves and metal forceps to eliminate human scent. Baskets were sanitized on high heat at least daily in a commercial dishwasher, without soap. All targets and distractors were handled by the same person to eliminate the dog’s ability to identify a single person associated with the target. Each arm of the scent wheel is identical to negate any visual cues that may enable detection capabilities. Dogs were monitored for characteristic changes in behavior related to detection of a target odor, including pausing and turning head abruptly at a position or emitting its trained final response of sitting at a target. All dogs were operated off lead by the handler. The dogs where handled by a Lead Canine Instructor who had 6 years of canine training and handling experience. This canine instructor did assist in training the dogs for this project. The handler was blinded to the target location and upon releasing the dog into the room, the handler stood at the door and stared straight ahead to avoid influencing the dog. If the dog indicated on a basket, the test moderator who was in the test room told the handler to reward or withhold the reward. This process insured that the dogs were not rewarded for indicating on a distractor virus and then being subsequently imprinted on the distractor virus because it received its reward. Blank trials contained eight distractors and no BVDV. Blank trials were utilized to insure that the dogs did not find a live target every time they searched the scent wheel, which reduces the propensity of the dog to alert to the final position regardless of whether it contains a target.

The dogs were taught to search position 1 to position 8 and then exit the room. The test moderator at the end of the trial notated the number of distractors that the dog searched in each trial. This provided the ability to calculate the total number of distractors searched by the dog.

### Data Analysis

Search results for each trial were recorded and entered into a spreadsheet (Microsoft Excel, Microsoft, Redmond, WA, USA). The sensitivity, specificity, and associated 95% confidence intervals were calculated using an online calculator (http://vassarstats.net).

## Results

The results for this study are shown in Table 2. Dog 1 alerted to 28 of 34 total target presentations and emitted 6 alerts across a total of 317 distractor presentations. Dog 2 alerted to 30 of 31 total target presentations and emitted 2 alerts across a total of 287 distractor presentations. The diagnostic sensitivity of Dog 1 [0.850, 95% confidence interval (CI): 0.701–0.942] was lower than that of Dog 2 (0.967, 95% CI: 0.837–0.994), and both dogs exhibited very high diagnostic specificity (0.981, 95% CI: 0.960–0.993) and (0.993, 95% CI: 0.975–0.999), respectively.

**Table 2:** **Individual dog results**.

	Dog 1	Dog 2
Sensitivity	0.85	0.967
Specificity	0.981	0.993
Total number of search past targets (i.e., misses)	6	1
Total number of positive indications	28	30
Total number of positive trials	34	31
Total number of blank trials (i.e., no BVDV present)	24	20
Total number of negative samples searched	317	287
Total number of false indications	6	2

A total of 65 positive and 44 blank (i.e., no BVDV present) trials were conducted. Each trial had a possibility of up to seven or eight distractors. There were 109 total trials in the study and after back calculating for the total number of distractors experienced, there were 604 distractors encountered by the dogs. There were eight total false responses that occurred while discriminating the 604 distractors used during this study. This demonstrates that the dogs did discriminate a large number of distractors and maintained a high rate of specificity. The dogs did not alert on seven targets and false responded on eight distractors. No search past target was attributed to a specific target (i.e., type of BVDV, see Table 1). Data for false responses were not collected for a specific distractor; therefore, it is unknown if the dogs false responded on any one distractor. Observations made by the test moderator were that the dogs false indicated on multiple distractors.

## Discussion

We hypothesized that dogs were able to detect and differentiate BVDV-infected cell cultures utilizing odors associated with different VOC expression patterns. Several recent reports indicate that cellular infections with viruses are associated with unique profiles of VOC (7–10, 17), but no published studies equate these VOC with an odor that is detectable by trained dogs. In this study, we were able to train dogs to discriminate cell cultures infected with BVDV from uninfected cell cultures and from cell cultures infected with one of two other viruses demonstrating the potential to detect the presence of odors associated with a target virus. The detection and discrimination of infected cell cultures by trained dogs supports our hypothesis of a unique odor profile associated with BVDV infection. As detection by the dog is immediate, this method has potential for use in real-time detection of viral pathogens in field situations.

Biochemical mechanisms underlying the release of disease-related VOC are largely unknown (4, 5, 12). No published studies define which, if any, VOC are detectable as an odor by dogs and the identity of the substances which alerted our dogs to infection is speculative. It is possible that VOC profiles associated with viral infections are analogous to the odor profile detected by our dogs. Previous studies demonstrated that compounds induced by pathogen infection may be detected as an odor (11), but current chemical analytical techniques have not defined the full odor profile which would control the alerting behavior of a dog.

Our study utilized purpose-bred detection dogs and training methods proven to be successful for the detection of other target substances, such as explosives. To the best of our knowledge, no previous attempts to detect and discriminate viral targets utilizing trained dogs have been made. The sensitivity and specificity of detection in the current study are similar to studies using dogs to detect the presence of neoplastic disease. Two studies evaluating the ability of dogs to detect prostate cancer-associated compounds in urine have yielded conflicting results. Elliker et al. (18) reported that trained dogs did not generalize on a prostate cancer odor and did not alert to new cancer samples following training on 50 unique prostate cancer samples and 67 controls. However, this study did not use purpose-bred detection dogs and the dogs lacked any previous detection experience. By contrast, Cornu et al. (19) trained a single dog to alert to positive prostate cancer samples with a sensitivity of 0.91. The differing results suggest that training methods, individual canine capabilities, and trial evaluations can affect outcomes and must be rigorous and scientifically sound to allow valid conclusions.

Results of our study suggest that trained dogs have the potential to function as a highly sensitive, highly specific, and mobile sensory technology to detect pathogen targets. Trained dogs could bridge the gap in pathogen and disease detection until other technologies are developed. The use of trained detector dogs has proven to be successful as a real-time mobile sensory technology in many inclement environments in the world during military, police, rescue, and other operations. Conversely, current analytical technologies capable of detecting diseases or pathogens in real-time in operational scenarios are unavailable, and there is no reported technology that can follow odor to its source like a dog.

Limitations of the current study include the use of cell culture-derived BVDV, use of a small number of trained dogs, a single-blind experiment, and the completion of the experiment in a climate-controlled environment. We did not evaluate the lowest level of detection of BVDV; however, our 0.5 ml samples of cell cultures were well within the range of detection by the dogs. We do not know at what level the sample becomes undetectable. Future research should focus on multiple target viruses, utilize a greater number of trained canids, and include a greater number of distractors. The test moderator was in the BSL2 with the dog making this a single-blind experiment. It is not known if the dog or handler was influenced by the moderator’s knowledge of the target position and how that may/may not have influenced the results. The dogs were off lead and the only command given by the handler was to “come” (i.e., out of the BSL2 room). Future projects should be conducted in a double-blind fashion. Virus detection should be conducted in different environmental conditions to assess the effects of temperature and humidity on sensitivity and specificity. The ability of dogs trained on cell culture-derived BVDV to correctly identify samples collected from infected hosts should be explored to determine if dogs can successfully detect viral infection utilizing a variety of sample types (e.g., breath, nasal discharge, sweat, and saliva).

The potential for detector dogs or their handlers becoming infected or harmed by a potential pathogen or transmitting the pathogen should be considered. Specific target viruses selected for study should be evaluated for risk to the detector dogs or transmission to humans, animals, and plants.

## Conclusion

Our study indicates that dogs can detect and discriminate virus-infected cell cultures. Apparently, unique odors associated with viral infections allow the dogs to obtain high rates of sensitivity and specificity. This finding demonstrates the potential for utilizing dogs to detect pathogens in real-time, which would be useful to identify or contain pathogen outbreaks, deter acts of bioterrorism, facilitate immediate treatment and containment of pathogen outbreaks, and reduce the need to transport samples for laboratory testing. Given that this demonstrated capability of dogs to detect odors associated with viruses and act as a RMST, additional research and development are warranted. In addition, dogs offer a platform for discovery to advance disease and chemical sensing machine technologies. Dogs provide a three order of magnitude more sensory capacity than most current diagnostic instruments. Therefore, they could be used for identifying specific VOC biomarkers related to disease which could aid metabolomics discoveries in other fields, such as analytical chemistry and wildlife biology.

### Manufacturer Information

a Corning^®^ cell culture flask, Corning Inc., Corning, NY, USA.
b GIBCO^®^ MEM, 10×, 11430, GIBCO by Life Technologies Corp., Grand Island, NY, USA.
c Seradigm, Providence, UT, USA.
d HyClone Laboratories Inc., Logan, UT, USA.
e GIBCO^®^ l-glutamine (200 mM), GIBCO by Life Technologies Corp., Grand Island, NY, USA.
f GIBCO^®^ Antibiotic-Antimycotic (100×), GIBCO by Life Technologies Corp., Grand Island, NY, USA.
g GIBCO^®^ Sodium Bicarbonate 7.5% solution, GIBCO by Life Technologies, Grand Island, NY, USA.
h Gentamicin sulfate powder, AMRESCO, Solon, OH, USA.

## Author Contributions

CA, TP, and PW developed the research idea and experimental design. TP and PG propagated the viruses and prepared training and testing aids. TF and BR performed canine training and testing. CA, TP, PW, and HM co-wrote the manuscript and all authors read and approved the final manuscript.

## Conflict of Interest Statement

The authors declare that they have no conflicts of interest. There are no MTAs, patents, or patent applications that apply to reagents, methods, or data in the paper.
